# Cortical thinning and Cognition in Cerebral Small Vessel Disease

**DOI:** 10.1002/alz.086495

**Published:** 2025-01-03

**Authors:** Alifiya Kapasi, Melissa Lamar, Maude Wagner, Arnold M Evia, David A. Bennett, Sue E. Leurgans, Konstantions Arfanakis, Julie A. Schneider

**Affiliations:** ^1^ Rush Alzheimer’s Disease Center, Chicago, IL USA; ^2^ Rush Alzheimer’s Disease Center, Rush University Medical Center, Chicago, IL USA; ^3^ Rush University Medical Center, Chicago, IL USA

## Abstract

**Background:**

Evidence is accumulating that cerebral small vessel disease may contribute to neurodegeneration. Brain arteriolosclerosis, a prominent type of small vessel disease in the aging brain, is associated with cognition. Using a previously developed published automated in‐vivo MRI classifier for arteriolosclerosis (ARTS), we examined cross‐sectionally the associations between ARTS, cortical thinning, and cognition.

**Method:**

Data came from 1,023 older participants from 5 ongoing Rush Alzheimer’s Disease Center cohort studies. Participants underwent detailed annual cognitive evaluations and completed at least one in‐vivo 3T MRI scan and one cognitive evaluation during follow‐up. In addition to global cognitive measures and MRI‐derived measures for ARTS score, data including global and lobar cortical thinning composites calculated using FreeSurfer. Using the latest completed MRI scan and a close assessment of cognition, linear regression models were employed to examine the association of ARTS with cortical thinning (global and regional) and global cognition. Further, mediation analyses were used to examine whether cortical thinning mediates the relationship between ARTS and global cognition. Models were all adjusted for age, sex, education, vascular risk factors, and scanner site.

**Result:**

Participants had an average age at 80 years (SD = 7) at last MRI scan, and 79% were women. Higher ARTS score was associated with global cortical thinning (estimate = ‐0.14; SE = 0.02, p<0.001), as well as regional cortical thinning in all four lobes (all p’s >0.017). Higher ARTS score was associated with lower global cognition (estimate = ‐0.49; SE = 0.10, p<0.001). In mediation analyses, cortical thinning accounted for 35% of the association between ARTS and global cognition.

**Conclusion:**

In vivo MRI‐derived measures for cerebral arteriolosclerosis are associated with global and lobar cortical thinning. The association between ARTS and cognition is partially mediated by cortical thinning. Findings suggest that cerebral small vessel disease may contribute to neurodegeneration and, both directly and indirectly, to subsequent cognitive impairment.

